# Circular RNA FNDC3B Protects Oral Squamous Cell Carcinoma Cells From Ferroptosis and Contributes to the Malignant Progression by Regulating miR-520d-5p/SLC7A11 Axis

**DOI:** 10.3389/fonc.2021.672724

**Published:** 2021-08-09

**Authors:** Jie Yang, Xing-Hua Cao, Ke-Feng Luan, Yun-Dong Huang

**Affiliations:** ^1^Department of Stomatology, Affiliated Hospital of Weifang Medical University, Weifang, China; ^2^Department of Stomatology, The Second People’s Hospital of Weifang, Weifang, China

**Keywords:** oral squamous cell carcinoma, ferroptosis, circFNDC3, miR-520d-5p, SLC7A11

## Abstract

Oral squamous cell carcinoma (OSCC) is a common head and neck malignancy with increasing mortality and high recurrence. Ferroptosis is an emerging programed cell death and plays an essential role in tumorigenesis. Circular RNAs (circRNAs) have been reported as a type of critical regulators in OSCC development. In this study, we identified the function of circular RNA FNDC3B (circFNDC3B) in regulating ferroptosis during the malignant progression of OSCC. Our data demonstrated that the silencing of circFNDC3B by shRNA inhibited GPX4 and SLC7A11 expression and enhanced ROS, iron, and Fe^2+^ levels in OSCC cells. CircFNDC3B knockdown reinforced erastin-induced inhibitory effect on OSCC cells. The depletion of circFNDC3B repressed cell proliferation and enhanced cell apoptosis of OSCC cells. Mechanically, circFNDC3B was able to increase SLC7A11 by targeting miR-520d-5p. The overexpression of SLC7A11 reversed circFNDC3B depletion or miR-520d-5p-induced ferroptosis phenotypes of OSCC cells. Moreover, tumorgenicity assays in nude mice showed that the depletion of circFNDC3B repressed OSCC cell growth *in vivo*. Taken together, we concluded that circFNDC3B attenuated ferroptosis of OSCC cells and contributed to OSCC progression by regulating the miR-520d-5p/SLC7A11 axis. CircFNDC3B, miR-520d-5p, and SLC7A11 may serve as potential therapeutic targets of OSCC.

## Introduction

Oral squamous cell carcinoma (OSCC) is a prevalent head and neck malignancy that occurs in oropharynx and mouth (1). OSCC serves as life-threatening cancer that 8.0%–8.5% of men and 4.0–8.1% of women may suffer from OSCC (2). The 5-year survival incidence of OSCC patients remains low and current therapeutic strategies are still unsatisfactory due to the metastasis (3, 4). Moreover, ferroptosis, as a programmed cell death described by the iron metabolism and ROS accumulation, plays crucial roles in cancer development and has become the therapeutic targets for various cancers (5–7). However, the function of ferroptosis and the molecular mechanism in OSCC progression remains unclear.

Non-coding RNAs have been widely identified to play various roles in the pathogenesis of OSCC (8). Circular RNA (circRNAs) is a sort of non-coding RNA with closed-loop structure (9). Moreover, circRNAs have been reported to have several crucial functions, such as protein binding and miRNA sponge (10–12). MiRNA sponge is the most commonly identified role of circRNAs in cancer development (10–12). CircRNAs share binding sites with miRNAs, and they compete with one another to act as competing endogenous RNAs (ceRNAs) to further regulate targeted genes in cancer progression (10–12). CircRNAs have become the biomarkers of cancer diagnosis and prognosis (13, 14). Several circRNAs have been reported to participate in the regulation of OSCC development (15, 16). Meanwhile, circular RNA FNDC3B (circFNDC3B) has been reported in the regulation of cancer cell proliferation, apoptosis, and migration in multiple malignancies, such as esophageal cancer, bladder cancer, and colon cancer (17–19). However, the effect of circFNDC3B on OSCC is still obscure. It has shown that circRNAs can exert function by wording as competing endogenous RNA (ceRNA) of microRNAs, which are another type of small non-coding RNAs. MiRNAs play essential roles in the development of OSCC by modulating various processes in the tumorigenesis, such as autophagy, invasion, apoptosis, and proliferation (20, 21). Furthermore, miR-520d-5p serves as a cancer suppressor in multiple malignancies, including cervical cancer, liver cancer, and lung cancer (22–24), but the relationship of miR-520d-5p with OSCC remains elusive. Meanwhile, solute carrier family 7, membrane 11 (SLC7A11), a 12-pass transmembrane protein, is a key component of the amino acid transporter system xc^–^ and a critical negative regulator of ferroptosis (25, 26).

In this study, we tried to explore the function of circFNDC3B in OSCC and we found that circFNDC3B attenuated ferroptosis of OSCC cells and contributed to OSCC progression by regulating the miR-520d-5p/SLC7A11 axis.

## Materials and Methods

### Cell Culture

The CAL27 and SCC15 cell lines were cultured in fetal bovine serum (10%, HyClone, USA)-supplemented DMEM medium (Thermo, USA) containing 1% penicillin/streptomycin (Gibco, USA) at 37°C and 5% CO_2_. The circFNDC3B shRNA (RiboBio, China), miR-520d-5p mimic/inhibitor (RiboBio, China) were synthesized and purchased. The exogenous circFNDC3B and SLC7A11 were fully synthesized and purchased (GenScript, China). The transfection in the cells was performed using Lipofectamine 3000 (Invitrogen, USA) according to the instruction.

### Clinical Tissues

The clinical OSCC samples (n=32) were collected from the patients underwent surgical resection without no radiotherapy or chemotherapy in the Affiliated Hospital of Weifang Medical University (No. 2428 Yuhe Road, Kuiwen District, Weifang, Shandong Province, China). The expression and the correlation of circFNDC3B, SLC7A11, and miR-520d-5p were analyzed in the tissues. The application of the samples was under the informed consent from the patients. The experiment was approved by the Ethics Committee of the Affiliated Hospital of Weifang Medical University.

### Western Blot Analysis

The CAL27 and SCC15 cells with the indicated treatment and tumor tissues (tumor tissue was ground using a tissue grinder) from tumorigenicity analysis in the nude mice were isolated by RIPA reagents supplemented with 1% proteinase inhibitor (Sigma, USA) and quantified by BCA Kit (Sigma, USA). The samples were subjected into SDS-PAGE gel electrophoresis (12%) and transferred into PVDF membranes (Millipore, USA), blocking using nonfat milk (5%) at 25°C for 2 hours and incubating with primary antibodies overnight at 4°C. The samples were further incubated using secondary antibodies at 25°C for 1.5 hours at room temperature and detected using enhanced chemiluminescence solution (Millipore, USA). The antibodies information was shown as follows: SLC7A11 (ab175186, Abcam, USA), β-actin (ab8226, Abcam, USA), GPX4 (ab125066, Abcam, USA), second antibodies (ab216773 (Goat anti-Rabbit), ab216772 (Goat anti-Mouse), Abcam, USA).

### Analysis Iron and ROS Levels

The iron and reactive oxygen species (ROS) levels were analyzed as the previous reports (27, 28). DCFH-DA staining (Sigma, USA) was applied to detect ROS levels and Iron Assay Kit (Sigma, USA) was applied to measure iron and Fe^2+^ levels.

### CCK-8

The Cell Counting Kit-8 assays (CCK-8, Promega, USA) were applied to analyze the viabilities of CAL27 and SCC15 cells. CAL27 and SCC15 cells (2 × 10^3^/well) were seeded into 96-well plates and cultured for 24 hours, 48 hours, 72 hours, and 96 hours. CCK-8 reagents were added in the cells and the cells were cultured for 2 hours at 37°C. The results were analyzed using a microplate reader (BioTek, USA) at 450 nm.

### Colony Formation Assays

The CAL27 and SCC15 cells (5 × 10^3^/well) were put in 6-well dishes and cultured in DMEM at 37°C. After two weeks, cells were cleaned with PBS Bufferand fixed using formaldehyde (1%), and dyed with crystal violet dye at the dose of 1%, after which the colonies were captured and observed *via* a microscope (Olympus, Japan). The colony numbers were calculated by using the ImageJ software.

### Apoptosis Analysis

After the indicated treatment of 48 hours, 1×10^6^ CAL27 and SCC15 cells were collected and fixed by paraformaldehyde (4%) at 25°C for 15 minutes and re-collected using the binding buffer. 5 µl propidium iodide (CST, USA) and 10 µl Annexin V-FITC (CST, USA) were incubated with the samples in the dark for 15 minutes. The results were detected using FACS Caliber (BD Bioscience, USA) and analyzed by applying Flowjo 7.6.1. The apoptosis was represented by AnnexinV+ (Q2 and Q3). The representative images of flow cytometry analysis were shown and the quantitative analysis from three repeated experiments was shown in histogram graph.

### Quantitative Reverse Transcription-PCR (RT-qPCR)

Total RNAs were extracted from cells and tumor tissues using TRIZOL (Sigma, USA) and cDNA reverse transcription was performed (Thermo, USA). The RT-qPCR assays were carried out using SYBR-Green (Vazyme, China). The data was normalized to GAPDH expression and calculated by a formula of 2^-ΔΔCt^. The primer information was shown as follows: circFNDC3B forward TCACAATAAGAGCAGAGGATGG; circFNDC3B reverse GGCAGTTCCAGAGGGATTT; miR-520d-5p CTACAAAGGGAAGCCCTTTC; GAPDH forward TGACGTGCCGCCTGGAGAAC; GAPDH reverse CCGGCATCGAAGGTGGAAGAG; U6 forward CTCGCTTCGGCAGCACA; U6 reverse AACGCTTCACGAATTTGCGT.

### Luciferase Reporter Gene Assay

The luciferase activity analysis was performed using Dual-Luciferase Reporter Assay Kit (Promega, USA). The CAL27 and SCC15 cells co-treated with miR-520d-5p mimic and pGL4.17-circFNDC3B or pGL4.17-SLC7A11 mRNA 3’ UTR by using riboFECT™ CP Transfection Kit (RiboBio, China), followed by the analysis of luciferase activities based on the Dual-luciferase Reporter Assay System (Promega, USA) after 48 hours of treatment. The luciferase activities of Renilla were measured as a control.

### Tumorigenicity Analysis

The tumorigenicity analysis was conducted in BALB/c nude mice (6-weeks-old, male). The mice were maintained at pathogen-free condition. The 5 × 10^6^ CAL27 cells were treated with control shRNA or circFNDC3B shRNA and subcutaneously injected into the nude mice (N = 5). The mice were sacrificed after 30 days and the tumor volume was calculated using the formula of (length × width^2^)/2. The tumor tissues were collected and weighted. All experiments were conformed to the guidelines of Animal Ethics and welfare Committee of Affiliated Hospital of Weifang Medical University.

### Statistical Analysis

Data were expressed as mean ± SD, and the statistical analysis was conducted using GraphPad prism 7. The unpaired Student’s *t*-test was used to compare two groups, and the one-way ANOVA was used to compare among multiple groups. *P* < 0.05 was considered as statistically significant.

## Results

### CircFNDC3B Protects OSCC Cells Against Ferroptosis

**Figure 1:**
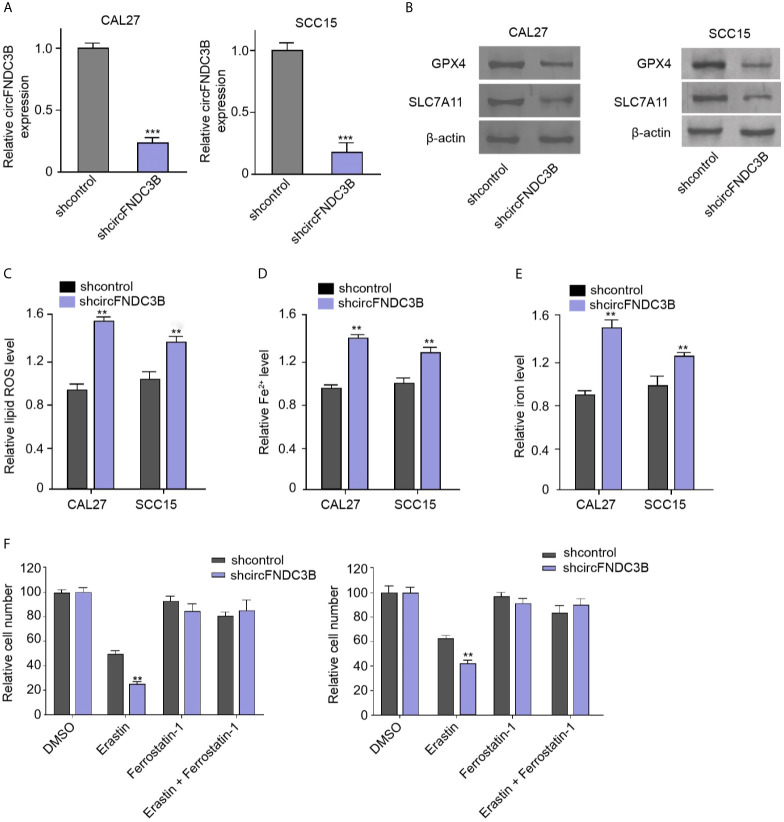
CircFNDC3B protects OSCC cells against ferroptosis. **(A–F)** The circFNDC3B shRNA treated the CAL27 and SCC15 cells. **(A)** The RT-qPCR analysis of circFNDC3B expression. **(B)** Western blot of GPX4 and SLC7A11 expression. **(C–E)** Analysis of ROS, iron, and Fe^2+^ levels. **(F)** CCK-8 assays of cell viability. N = 3, mean ± SEM: **P < 0.01, ***P < 0.001.

First, the effectiveness of circFNDC3B shRNAs was verified in the CAL27 and SCC15 cells (**Supplementary Figure 1A**). And then we validated that the depletion of circFNDC3B repressed the viability of CAL27 and SCC15 cells (**Supplementary Figure 1B**). The circFNDC3B shRNA-2 was selected in the subsequent experiments. We evaluated the effect of circFNDC3B on ferroptosis of OSCC cells. Consistent with the reduction of circFNDC3B expression, the silencing of circFNDC3B using shRNA repressed the levels of ferroptosis negative regulators, including GPX4 and SLC7A11, in CAL27 and SCC15 cells (**Figures 1A, B**). The circFNDC3B shRNA induced ROS, iron, and Fe^2+^ accumulation in CAL27 and SCC15 cells (**Figures 1C–E**). Moreover, erastin, a ferroptosis activator, inhibited CAL27 and SCC15 cell viability and circFNDC3B knockdown reinforced this effect in the model (**Figure 1F**), indicating that circFNDC3B protects OSCC cells against ferroptosis.

### CircFNDC3B Contributes to OSCC Cell Proliferation *In Vitro*

**Figure 2:**
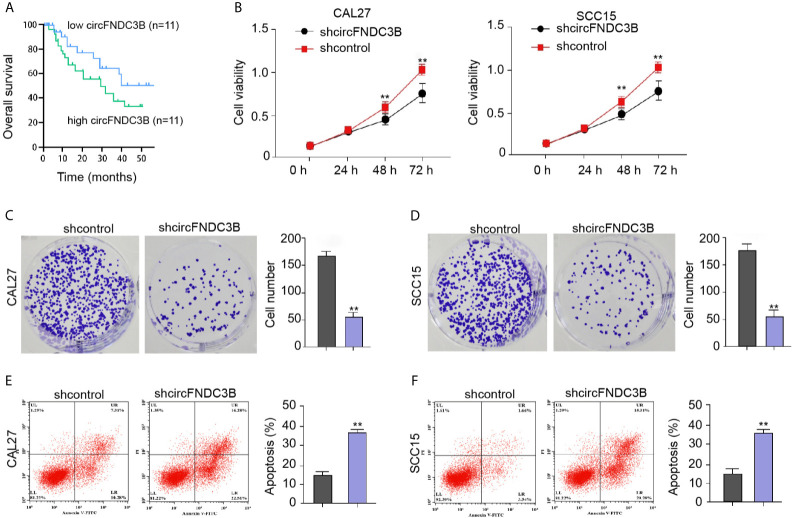
CircFNDC3B contributes to OSCC cell proliferation *in vitro*. **(A)** The expression of circFNDC3B was analyzed by qPCR in the clinical OSCC tissues and the OSCC samples were divided into two group according to the circFNDC3B expression. The correlation of circFNDC3B with the prognosis of OSCC patients was evaluated by the overall survival analysis. **(B–F)** The circFNDC3B shRNA treated the CAL27 and SCC15 cells. **(B)** CCK-8 assays of cell viability. **(C, D)** Colony formation assays of cell proliferation. **(E, F)** Flow cytometry analysis of cell apoptosis. N = 3, mean ± SEM: **P < 0.01.

Next, we observed that the expression of circFNDC3B in clinical OSCC tissues was negative correlated with the prognosis of the OSCC patients (**Figure 2A**). We then concerned about the effect of circFNDC3B on OSCC cell proliferation *in vitro*. We found that the circFNDC3B shRNA attenuated cell viability in CAL27 and SCC15 cells (**Figure 2B**). The colony formation numbers of CAL27 and SCC15 cells were repressed by circFNDC3B silencing (**Figures 2C, D**). In addition, the circFNDC3B knockdown promoted CAL27 and SCC15 cell apoptosis, suggesting that circFNDC3B contributes to OSCC cell proliferation *in vitro* (**Figures 2E, F**).

**Figure 3:**
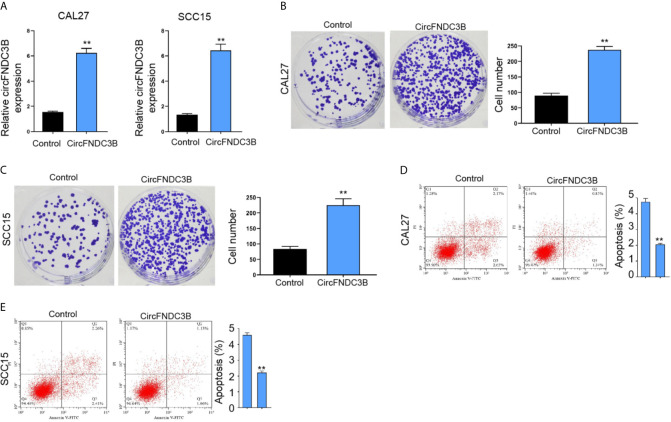
CircFNDC3B promotes OSCC cell proliferation *in vitro*. **(A–E)** The pcDNA3.1-circFNDC3B overexpressing vectors treated the CAL27 and SCC15 cells. **(A)** CCK-8 assays of cell viability. **(B, C)** Colony formation assays of cell proliferation. **(D, E)** Flow cytometry analysis of cell apoptosis. N= 3, mean ± SEM: **P < 0.01.

Moreover, the overexpression effectiveness of circFNDC3B was confirmed in CAL27 and SCC15 cells (**Figure 3A**). The overexpression of circFNDC3B enhanced CAL27 and SCC15 cell proliferation (**Figures 3B, C**) and repressed CAL27 and SCC15 cell apoptosis *in vitro* (**Figures 3D, E**).

### CircFNDC3B Enhances SLC7A11 Expression by Targeting miR-520d-5p

**Figure 4:**
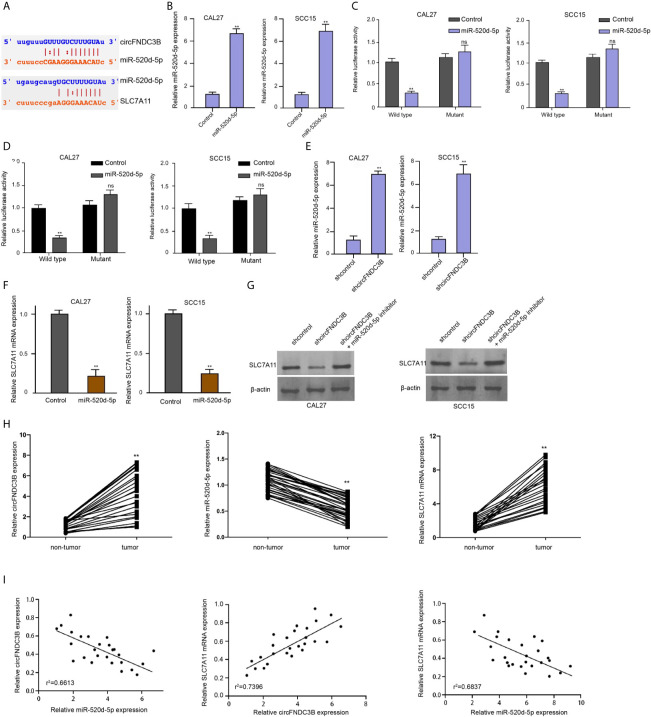
CircFNDC3B enhances SLC7A11 expression by targeting miR-520d-5p. **(A)** The interaction of miR-520d-5p with circFNDC3B and SLC7A11 was identified in ENCORI online database. **(B–D)** MiR-520d-5p mimic or control mimic was used to treat CAL27 and SCC15 cells. **(B)** The RT-qPCR analysis of miR-520d-5p expression. **(C, D)** Dual luciferase reporter assays of luciferase activities of circFNDC3B and SLC7A11 mRNA 3’UTR, respectively. **(E)** The RT-qPCR analysis of miR-520d-5p expression in CAL27 and SCC15 cells treated with circFNDC3B shRNA. **(F)** The RT-qPCR analysis of SLC7A11 expression in CAL27 and SCC15 cells treated with miR-520d-5p mimic. **(G)** Western blot analysis of SLC7A11 expression in CAL27 and SCC15 cells treated with circFNDC3B shRNA and miR-520d-5p inhibitor. N = 6, mean ± SEM: ns no significance, **P < 0.01. **(H)** The expression of circFNDC3B, SLC7A11, and miR-520d-5p was analyzed by qPCR in the clinical OSCC tissues and the related non-tumor tissues. **(I)** The correlation of circFNDC3B, SLC7A11, and miR-520d-5p was analyzed by qPCR in the clinical OSCC tissues. mean ± SEM: **P < 0.01.

We then identified the interaction of miR-520d-5p with circFNDC3B and SLC7A11 in ENCORI online database (**Figure 4A**). Consistent with the up-regulation of miR-520d-5p expression, the luciferase activities of circFNDC3B and SLC7A11 mRNA 3’UTR were repressed by miR-520d-5p mimic in CAL27 and SCC15 cells (**Figures 4B–D**). The silencing of circFNDC3B by shRNA enhanced miR-520d-5p expression and miR-520d-5p mimic reduced SLC7A11 expression in CAL27 and SCC15 cells (**Figures 4E, F**). Meanwhile, the SLC7A11 expression was attenuated by circFNDC3B shRNA, while miR-520d-5p inhibitor could rescue this result in the model (**Figure 4G**), suggesting that circFNDC3B enhances SLC7A11 expression by targeting miR-520d-5p. Clinically, we observed that the circFNDC3B and SLC7A11 expression was enhanced and miR-520d-5p expression was reduced in clinical OSCC tissues compared to the non-tumor tissues (**Figure 4H**). Meanwhile, miR-520d-5p was negatively correlated with circFNDC3B and SLC7A11 and circFNDC3B was positively correlated with SLC7A11 in clinical OSCC tissues (**Figure 4I**).

### CircFNDC3B Represses Ferroptosis of OSCC Cells by Inducing SLC7A11 Expression

**Figure 5:**
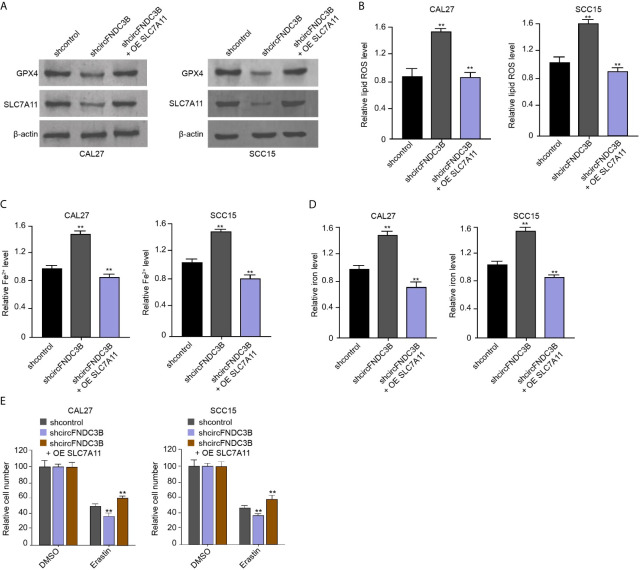
CircFNDC3B represses ferroptosis of OSCC cells by inducing SLC7A11 expression. **(A–E)** The circFNDC3B shRNA and pcDNA3.1-SLC7A11 were applied to co-treat CAL27 and SCC15 cells. **(A)** Western blot of GPX4 and SLC7A11 expression. **(B–D)** Analysis of ROS, iron, and Fe^2+^ levels. **(E)** CCK-8 assays of cell viability. N = 3, mean ± SEM: **P < 0.01.

We found that the silencing of circFNDC3B using shRNA repressed the levels of GPX4 and SLC7A11 in CAL27 and SCC15 cells, in which SLC7A11 overexpression rescued the expression of GPX4 and SLC7A11 (**Figure 5A**). The circFNDC3B shRNA enhanced ROS, iron, and Fe^2+^ accumulation in CAL27 and SCC15 cells, while SLC7A11 overexpression conversely regulated this effect in the model (**Figures 5B–D**). Moreover, erastin inhibited CAL27 and SCC15 cell viability and circFNDC3B knockdown reinforced this effect, in which SLC7A11 overexpression further reversed the effect of circFNDC3B knockdown in the model (**Figure 5E**), indicating circFNDC3B represses ferroptosis of OSCC cells by inducing SLC7A11 expression.

### miR-520d-5p Induces Ferroptosis of OSCC Cells by Repressing SLC7A11 Expression

**Figure 6:**
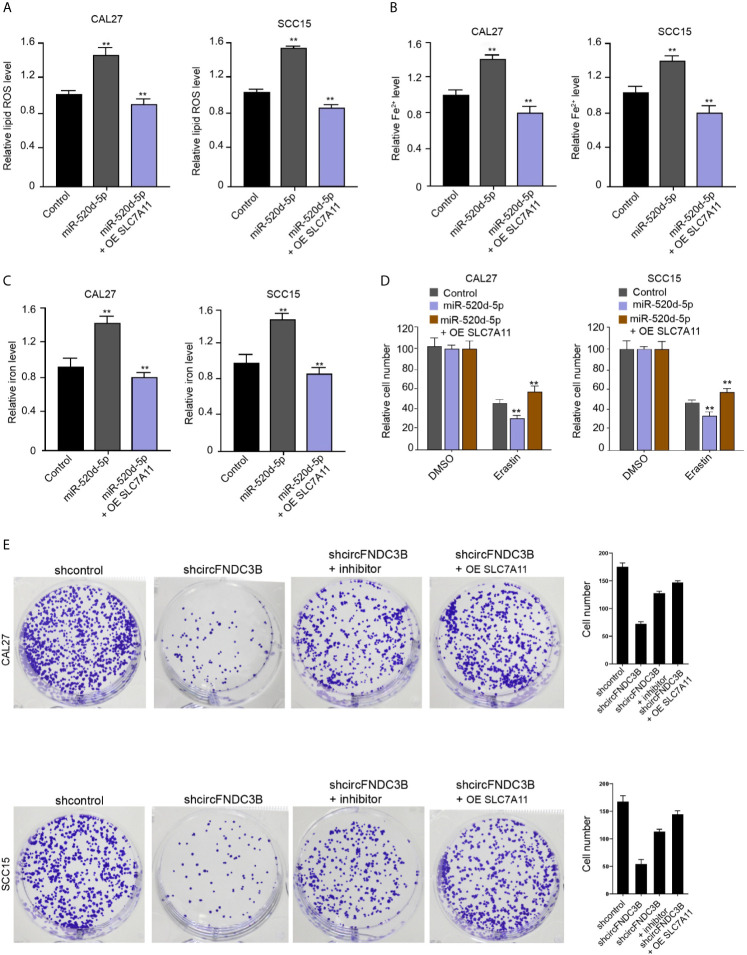
miR-520d-5p induces ferroptosis of OSCC cells by repressing SLC7A11 expression. **(A–E)** The miR-520d-5p mimic and pcDNA3.1-SLC7A11 were applied to co-treat CAL27 and SCC15 cells. **(A–C)** Analysis of ROS, iron, and Fe^2+^ levels. **(D)** CCK-8 assays of cell viability. **(E)** The CAL27 and SCC15 cells were treated with circFNDC3B shRNA or co-treated with circFNDC3B shRNA and miR-520d-5p inhibitor and pcDNA3.1-SLC7A11. Colony formation assays of cell proliferation. N = 3, mean ± SEM: **P < 0.01.

Our data showed that the treatment of miR-520d-5p mimic increased ROS, iron, and Fe^2+^ accumulation in CAL27 and SCC15 cells, while SLC7A11 overexpression conversely regulated this effect in the model (**Figures 6A–C**). Meanwhile, erastin reduced CAL27 and SCC15 cell viability and miR-520d-5p mimic enhanced this effect, in which SLC7A11 overexpression further reversed the effect of miR-520d-5p in the model (**Figure 6D**), implying that miR-520d-5p induces ferroptosis of OSCC cells by repressing SLC7A11 expression. Moreover, the colony numbers of CAL27 and SCC15 cells were repressed by the silencing of circFNDC3B, while the inhibitor of miR-520d-5p and SLC7A11 overexpression could reverse this effect (**Figure 6E**).

### CircFNDC3B Contributes to OSCC Cells Growth *In Vivo*

**Figure 7:**
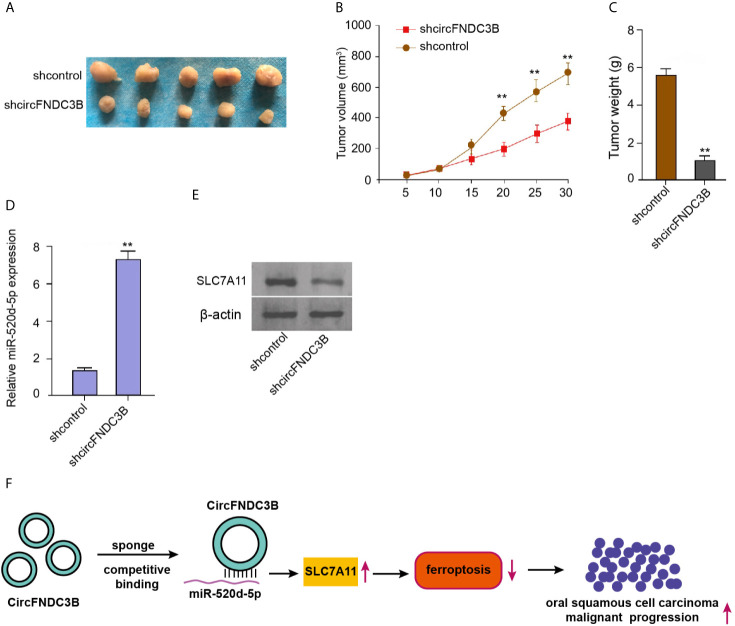
CircFNDC3B contributes to OSCC cells growth *in vivo.* **(A–E)** Tumorigenicity assays in nude mice injected with CAL27 cells, which were treated with control shRNA or circFNDC3B shRNA. **(A)** Representative images of dissected tumors. **(B, C)** The analysis of average tumor volume and tumor weight. (D) The RT-qPCR assays of miR-520d-5p expression. **(E)** Western blot analysis of SLC7A11 expression. **(F)** Schematic diagram of the proposed mechanism of circFNDC3B-mediated ferroptosis and progression of OSCC. N = 5, mean ± SEM: **P < 0.01.

Next, we validated the impact of circFNDC3B on OSCC cell growth *in vivo.* Tumorigenicity assays in nude mice showed that the silencing of circFNDC3B repressed the tumor size, tumor volume, and weight in the model (**Figures 7A–C**), suggesting that circFNDC3B contributes to OSCC cells growth *in vivo.* Besides, the miR-520d-5p expression was increased and SLC7A11 expression was decreased in the tumor tissues of circFNDC3B depletion group compared with the control group (**Figures 7D, E**).

## Discussion

OSCC is the prevailing women cancer affecting modern people and resulting in high mortality. Ferroptosis has been identified to play essential roles in cancer development and therapy. In this study, we uncovered that circFNDC3B protected OSCC cells against ferroptosis and promoted OSCC progression by regulating miR-520d-5p/SLC7A11 axis.

Previous studies have demonstrated the critical function of circFNDC3B in cancer development. It has been reported that circFNDC3B contributes to the invasion and migration by modulating CD44 and E-cadherin expression in gastric cancer cells (29). CircFNDC3B serves as a sponge of miR-937-5p to regulate TIMP3 expression and in colorectal cancer development (30). CircFNDC3B modulates renal cancer by down-regulating miR-99a expression (31). Several circular RNAs have been found to regulate OSCC progression. CircUHRF1 contributes to tumorigenesis of OSCC in a feedback loop-dependent manner (16). Circular RNA MMP9 promotes the metastasis of OSCC by regulating the mRNA stability of MMP9 (32). CircRNA_100290 modulates OSCC progression by targeting miR-378a/glucose transporter-1 (GLUT1)/glycolysis (33). Here, our data showed that silencing of circFNDC3B inhibited GPX4 and SLC7A11 expression and enhanced ROS, iron, and Fe^2+^ levels in OSCC cells. CircFNDC3B knockdown reinforced erastin-induced inhibitory effect on OSCC cells. The depletion of circFNDC3B repressed cell proliferation and enhanced cell apoptosis of OSCC cells. These data indicate the novel function of circFNDC3B in regulating ferroptosis during OSCC malignant progression, providing valuable evidence of the critical roles of circular RNAs in ferroptosis of cancer cells. We failed to observe that circFNDC3B affected the ferroptosis of normal oral mucosal HOMEC cells (data not shown). We observed that circFNDC3B affected both apoptosis and ferroptosis in OSCC cells. The correlation of circFNDC3B-meidated apoptosis and ferroptosis and which is the main effect induced by circFNDC3B need to be confirmed in further studies. Meanwhile, in this study, we identified that circFNDC3B was elevated in the clinical OSCC tissues. And we explored the function of circFNDC3B in OSCC cell ferroptosis by the knockdown of circFNDC3B using shRNA in the OSCC cells and we did not evaluate the effect of circFNDC3B by the overexpression of circFNDC3B. In the future investigation, the impact of circFNDC3B on OSCC cell ferroptosis should be confirmed.

Previous investigations have reported the function of miR-520d-5p in cancer development. MiR-520d-5p is a tumor-suppressor by repressing PTK2 in cervical cancer (24). MiR-520d-5p attenuates proliferation and promotes cell cycle arrest of glioma cells by inhibiting PTTG1 (34). MiR-520d-5p represses metastasis and tumor growth *via* targeting CTHRC1 in colorectal cancer (35). Many miRNAs participate in the regulatory network of OSCC pathogenesis. MicroRNA-504 represses invasion, migration, and proliferation of OSCC cells by regulating CDK6 (36). MicroRNA-625-3p contributes to migration of OSCC cells by targeting SCAI (37). MicroRNA−199a−5p induced a tumor suppression effect on OSCC by modulating NF−κB signaling (38). Meanwhile, it has been reported that SLC7A11 is a critical regulator of ferroptosis and metabolism (39, 40). It also has been found that miR-375/SLC7A11 signaling modulates proliferation and invasion of OSCC cells (41). In this study, we discovered that circFNDC3B induced SLC7A11 expression by serving as a ceRNA of miR-520d-5p and circFNDC3B/miR-520d-5p/SLC7A11 axis was involved in the regulation of ferroptosis-related phenotypes in OSCC cells. Our finding presents unreported mechanism involving circFNDC3B, miR-520d-5p, and SLC7A11 in attenuating ferroptosis of OSCC tumorigenesis, elucidating the correlation of circFNDC3B, miR-520d-5p, and SLC7A11 in OSCC development. Moreover, miR-520 is a huge miRNA family and in addition to miR-520d-5p, our bioinformatics analysis identified the potential interaction sites between circFNDC3B with other members of miR-520 family, such as miR-520h, miR-520g-3p, miR-520a-5p, miR-520c-3p, miR-520e, miR-520b, miR-520f-3p, miR-520d-3p, miR-520a-3p, miR-520c-5p, and miR-520g-3p. And in addition to miR-520d-5p, we also found the potential binding sites between SLC7A11 with other members of miR-520 family, such as miR-520g-3p, miR-520h, miR-520b, miR-520c-5p, miR-520f-3p, miR-520a-5p, miR-520d-3p, miR-520a-3p, and miR-520c-3p. We just selected miR-520d-5p as an example in this study. The correlation of other members of miR-520 family with circFNDC3B/SLC7A11 axis in OSCC development should be investigated in future studies. Many critical molecules, such as SLC7A11, GPX4, ACSL4, and p53, play essential roles in the modulation of ferroptosis in cancer development. In the mechanical exploration of this study, we just selected SLC7A11 as an example and more mechanism and their correlation and logic should be investigated in further experiments. Importantly, SLC7A11 may just one of the downstream factors of circFNDC3B and miR-520 in the modulation of OSCC development and other potential targets need to be explored.

Taken together, we concluded that circFNDC3B attenuated ferroptosis of OSCC cells and contributed to OSCC progression by regulating miR-520d-5p/SLC7A11 axis (**Figure 7F**). The clinical significance of circFNDC3B and miR-520d-5p as the potential therapeutic targets for the treatment of OSCC cell ferroptosis is needed to further investigate.

## Data Availability Statement

The original contributions presented in the study are included in the article/**Supplementary Material**. Further inquiries can be directed to the corresponding author.

## Ethics Statement

The animal study was reviewed and approved by Affiliated Hospital of Weifang Medical University.

## Author Contributions

All authors contributed to the study conception and design. Material preparation, data collection and analysis were performed by JY and X-HC. The first draft of the manuscript was written by K-FL, Y-DH and all authors commented on previous versions of the manuscript. All authors contributed to the article and approved the submitted version.

## Conflict of Interest

The authors declare that the research was conducted in the absence of any commercial or financial relationships that could be construed as a potential conflict of interest.

## Publisher’s Note

All claims expressed in this article are solely those of the authors and do not necessarily represent those of their affiliated organizations, or those of the publisher, the editors and the reviewers. Any product that may be evaluated in this article, or claim that may be made by its manufacturer, is not guaranteed or endorsed by the publisher.

## Supplementary Material

Supplementary Figure 1 The validation of the effectiveness of circFNDC3B shRNA. **(A)** The circFNDC3B shRNA treated the CAL27 and SCC15 cells. **(A)** The RT-qPCR analysis of circFNDC3B expression. **(B)** CCK-8 assays of cell viability. N = 3, mean ± SEM: ***P* < 0.01.
