# Supplementary figures and images for: Circular RNA FNDC3B Protects Oral Squamous Cell Carcinoma Cells From Ferroptosis and Contributes to the Malignant Progression by Regulating miR-520d-5p/SLC7A11 Axis
Source: Front Oncol. 2021 Aug 9;11:672724. doi: 10.3389/fonc.2021.672724 (PMC8382281; doi:10.3389/fonc.2021.672724)

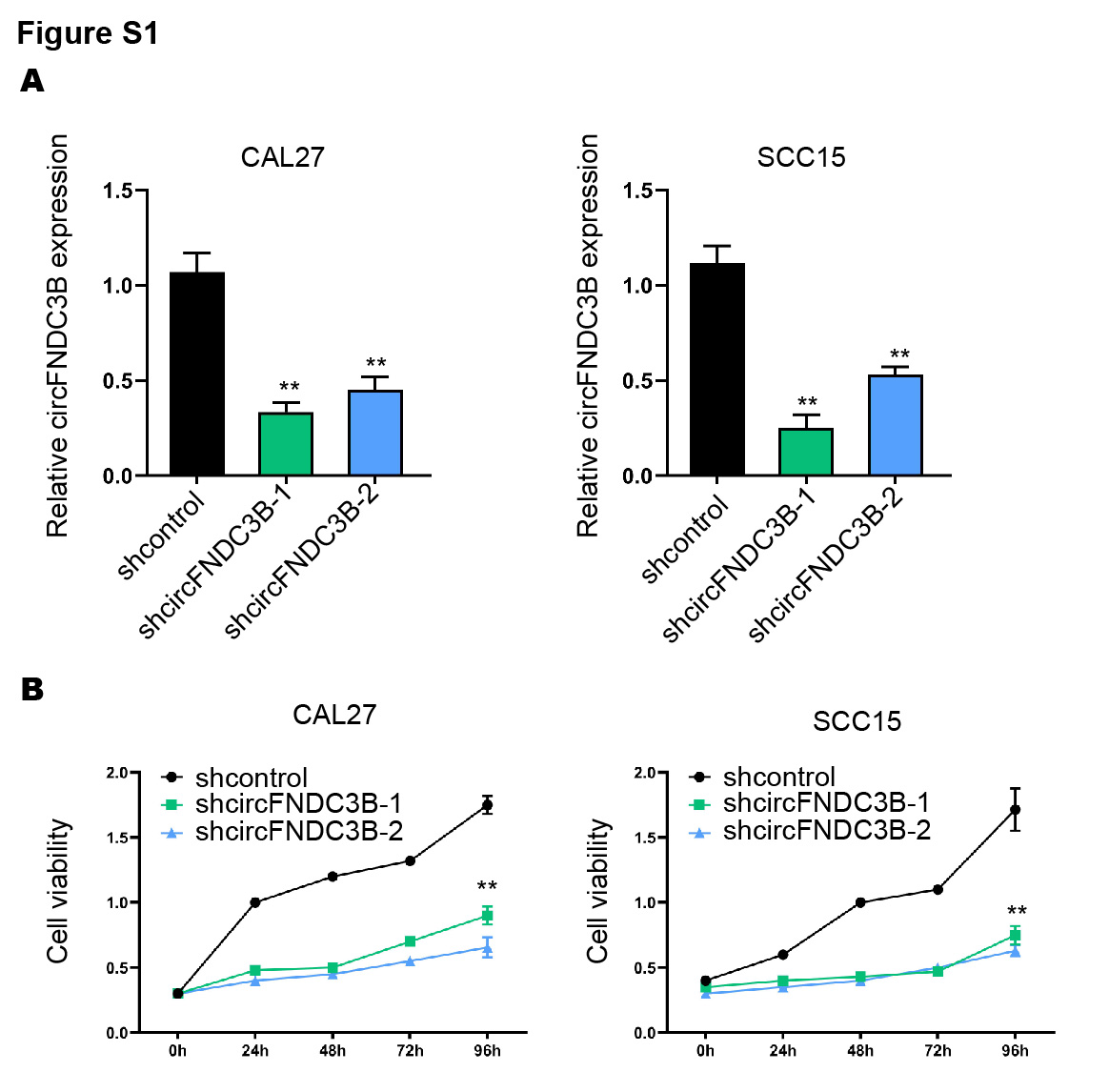

Supplement: Supplementary Figure 1 — The validation of the effectiveness of circFNDC3B shRNA. (A) The circFNDC3B shRNA treated the CAL27 and SCC15 cells. (A) The RT-qPCR analysis of circFNDC3B expression. (B) CCK-8 assays of cell viability. N = 3, mean ± SEM: **P < 0.01. [file Image_1.jpeg]
